# Suboptimal endoscopic cancer recognition in colorectal lesions in a national bowel screening programme

**DOI:** 10.1136/gutjnl-2018-316882

**Published:** 2019-12-10

**Authors:** Jasper L A Vleugels, Lianne Koens, Marcel G W Dijkgraaf, Britt Houwen, Yark Hazewinkel, Paul Fockens, Evelien Dekker, on behalf of the DISCOUNT study group, M C J M Becx

**Affiliations:** 1 Department of Gastroenterology and Hepatology, Amsterdam University Medical Center, location Academic Medical Center, Amsterdam, The Netherlands; 2 Department of Pathology, Amsterdam University Medical Center, location Academic Medical Center, Amsterdam, The Netherlands; 3 Clinical Research Unit, Amsterdam University Medical Center, location Academic Medical Center, Amsterdam, The Netherlands

**Keywords:** colonoscopy, colorectal cancer, colorectal cancer screening, endoscopy

## Message

The worldwide implementation of bowel cancer screening programmes (BCSPs) results in a growing number of early T1 colorectal cancers (T1 CRCs). Successful treatment of T1 CRCs starts with accurately recognising these lesions during endoscopy. This study performed in the Dutch BCSP showed that endoscopists correctly diagnosed T1 CRCs in only 39% of 92 cases (95% CI 30 to 49) and that this limited diagnostic accuracy of optical diagnosis resulted in different treatment outcomes. In patients with T1 CRCs that were optically not diagnosed as cancer and treated locally, adjuvant surgery was performed in 41% of cases, compared with 11% of patients with T1 CRCs that were correctly optically diagnosed (p=0.02).

## In more detail

In this prospective multicentre study (trial registration number: NTR4635, NCT02407925), endoscopists accredited for fecal immunochemical test (FIT)-positive colonoscopies within the Dutch BCSP were trained in optical diagnosis with our validated National Institute for Health and Care Excellence (NICE)-WASP (Workgroup serrAted polypS and Polyposis) module (online supplementary material 1 for full methods).^1 2^ A total of 27 endoscopists completed the training successfully and entered the prospective study, in which the endoscopists as well as pathologists reported their findings in a predefined structure. This facilitated high-quality data collection and ensured collection of exact data on all aspects of each detected lesion as location, size, Paris morphology, optical diagnosis (colorectal cancer (CRC), adenoma, hyperplastic polyp, sessile serrated lesion or other), endoscopic treatment method (biopsy, cold or hot snare polypectomy, endoscopic mucosal resection, biopsy for diagnosis or no treatment), completeness of resection and whether a tattoo was placed. Participating endoscopists recorded optical diagnosis using narrow band imaging in all consecutive FIT-positive colonoscopies for the Dutch BCSP during 1 year. Local treatment was defined as endoscopic resection or transanal endoscopic microsurgery. For all patients initially diagnosed with T1 CRCs, the original H&E staining slides were collected from local hospitals and reviewed. A specialist GI pathologist performed a blinded pathology review on the original slides to corroborate initial diagnosis and provide complete histological information on all T1 CRCs.^3–7^ Furthermore, for patients with suspected or established CRC, additional information on follow-up colonoscopies, adjuvant local or surgical therapy, and histopathological outcomes of adjuvant therapy was collected until January 2019.

10.1136/gutjnl-2018-316882.supp1 Supplementary data

**Figure 1:**
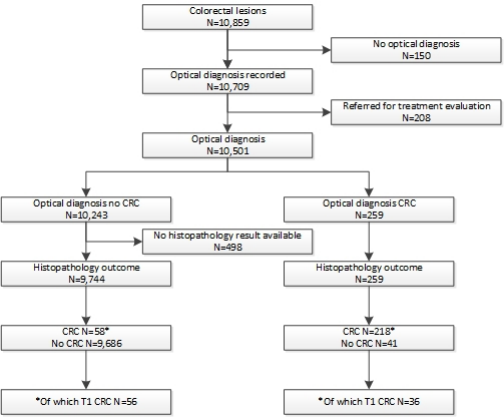
STARD flow chart describing study flow. Between February 2015 and February 2017, 28 participating endoscopists performed 3622 colonoscopies for the Dutch BCSP and detected 10 859 lesions during these colonoscopies. The figure shows the flow through the study along with the primary outcome of optical diagnosis of T1 CRC. Reasons for exclusion are noted. *Depicts the number of T1 CRCs of the total group of CRCs. BCSP, bowel cancer screening programme; CRC, colorectal cancer; STARD, Standards for Reporting Diagnostic Accuracy; T1 CRC, T1 colorectal cancer.

**Figure 2:**
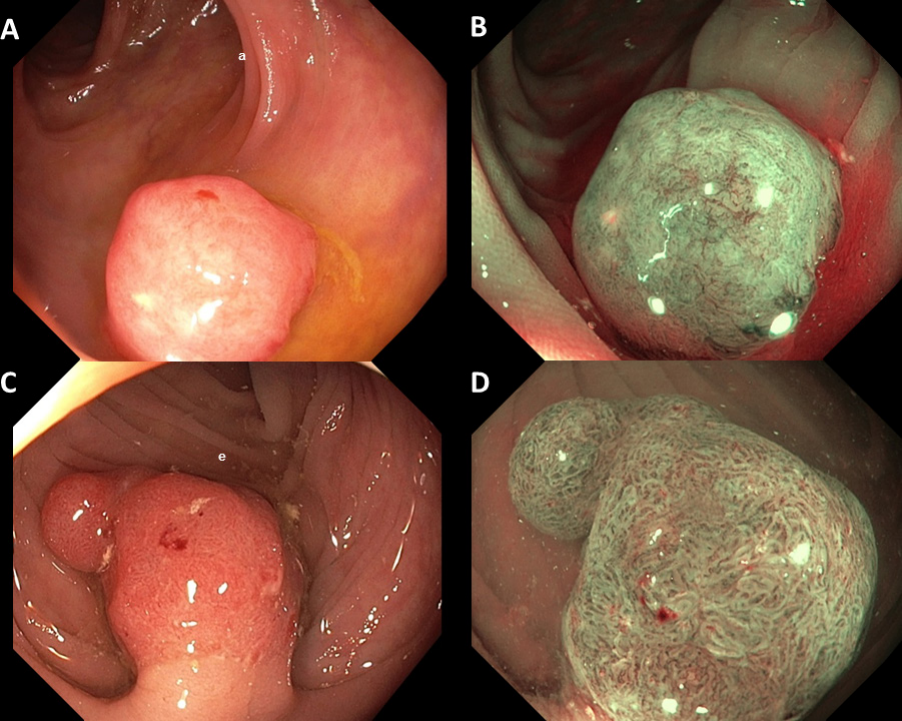
White light (A, C) and corresponding narrow band imaging (B, D) pictures of histologically confirmed T1 colorectal cancers optically diagnosed as adenomas.^27^

Between February 2015 and February 2017, 3622 colonoscopies for the Dutch BCSP were performed. Optical diagnosis and histopathology outcome were available for 10 004 lesions (figure 1). In total, 274 patients were diagnosed with CRC, including 90 patients with 92 T1 cancers (online supplementary table 1). The sensitivity for optical diagnosis of CRC was 79.0% (95% CI 73.7% to 83.6%), while the negative predictive value was 99.4% (95% CI 99.3% to 99.5%). Diagnostic test accuracies stratified for confidence level of optical diagnosis can be found in online supplementary table 2. Of 92 T1 cancers, 36 (39.1%, 95% CI 29.1 to 49.9) were correctly optically diagnosed as cancer. Of 56 T1 CRCs optically not diagnosed as cancer (figure 2), 11 (20%) were resected in a piecemeal fashion. In 38 patients, an additional colonoscopy had to be performed to mark the previous endoscopic resection site of the T1 cancer after histology outcome. Adjuvant oncological surgery after local treatment was performed for 2 of 18 (11%) T1 CRCs correctly optically diagnosed as cancer compared with 22 of 54 (41%) of those that were not (p=0.02). Overall, direct or adjuvant surgical treatment after local treatment was performed in 20 of 36 (56%) correctly optically diagnosed T1 CRCs and 24 of 56 (43%) T1 CRCs that were not optically diagnosed as cancer (p=0.29). More details can be found in online supplementary table 3. In 84 of these 90 patients, a median of 2 (IQR 1–2) follow-up exams were performed during a median endoscopic follow-up of 16 months (IQR 12–24). Three patients refused follow-up colonoscopies and three patients were lost to follow-up. One patient died from metastasised T1 CRC 12 months after initial diagnosis, and another patient with metastasised T1 CRC is still alive. None of the patients had a local recurrence. There was no difference in terms of survival and treatment outcomes between the optically correctly and incorrectly diagnosed T1 CRCs.

10.1136/gutjnl-2018-316882.supp2 Supplementary data

10.1136/gutjnl-2018-316882.supp3 Supplementary data

10.1136/gutjnl-2018-316882.supp4 Supplementary data

## Comments

BCSPs aim to detect CRC at an early stage. In a recent report of the Dutch FIT-based BCSP, 40% of all CRCs were T1.^8^ Patients with T1 cancers with favourable histological characteristics are at low risk for lymph node metastasis (6%–27%),^9–11^ and the majority of these lesions can be cured with endoscopic treatment.^12–15^ Several histological characteristics predict the risk of lymph node metastases.^4 5 10 16^ These risk factors can only be assessed when the lesion is resected en bloc. Therefore, successful treatment of these lesions starts with suspecting a T1 cancer, either with superficial submucosal (SMs) or deep submucosal (SMd) invasion.

Our study demonstrates that optical diagnosis of T1 CRC among accredited endoscopists in the Dutch BCSP can be further improved. Overall sensitivity for optical diagnosis of all stages of CRC was 79.0%, while the negative predictive value was 99.4%. This suboptimal sensitivity relied heavily on a limited sensitivity of optical diagnosis of T1 CRCs, which were optically diagnosed correctly as cancer in only 39.1% (95% CI 29.1 to 49.9). The limited diagnostic accuracy also resulted in suboptimal treatment decisions, as 20% of T1 CRCs optically not diagnosed as cancer were removed in a piecemeal fashion, thus prohibiting optimal histological analysis. As a result, adjuvant surgical treatment after local treatment was more frequently indicated and performed for patients with T1 CRCs that were not correctly optically diagnosed (41% vs 11%, p=0.02). However, in terms of survival and treatment outcomes, there were no differences between the two groups, although the median follow-up was limited to 16 months. Moreover, patient characteristics and preferences may have influenced the decision for adjuvant surgical treatment.

In a recently published prospective Spanish study in which 58 endoscopists from community and university hospitals examined over 2000 lesions of at least 10 mm in size using the NICE classification, the NICE classification identified lesions with SMd with 58% sensitivity (95% CI 48 to 69) and negative predictive value of 98% (95% CI 97 to 99).^17^ In another real-time Dutch study with endoscopists trained in optical diagnosis of CRC, which included large non-pedunculated lesions of >20 mm, a much higher sensitivity for optical diagnosis was reported, namely 79% (95% CI 64 to 89).^18^ However, the positive predictive value in this study was quite low at 69% (95% CI 57 to 78), which resulted in unnecessary surgery. This was also shown in other studies.^19 20^ Hence, we can conclude that incorrect optical diagnosis for predicting SM and SMd invasion results in suboptimal use of endoscopic and surgical treatment options, resulting in both overtreatment and undertreatment. On the other hand, there is a narrow gap between T1 cancer detected and diagnosed by endoscopy that is curable by endoscopic treatment methods. In most series, up to 40% of patients with T1 cancers resected by endoscopic mucosal resection, endoscopic submucosal dissection or endoscopic full-thickness resection have to undergo additional surgery anyway due to high-risk histological criteria.^21–23^

Observational studies with validated training in optical diagnosis of T1 cancers for community gastroenterologists are needed, aiming to achieve high sensitivity and negative predictive value for endoscopic prediction of T1 cancers. Preferably, endoscopists would also achieve high accuracies for differentiating between SMs and SMd. To determine whether lesions are suitable for endoscopic resection, a structured lesion assessment seems useful. The recent British Society of Gastroenterology and European Society of Gastrointestinal Endoscopy guidelines suggest to use the NICE and/or Kudo classification with high-definition virtual chromoendoscopy techniques to assess SM invasion, as these have shown good interobserver agreement and are easily adapted in clinical practice.^24 25^

This study has several limitations. We included all detected lesions, irrespective of size and morphology, for calculating diagnostic test accuracies. As the prevalence of CRC within small lesions is low, the negative predictive value of optical diagnosis of CRC is likely overestimated. On the other hand, every colorectal lesion can harbour CRC and should therefore be characterised before applying treatment. Furthermore, we excluded lesions that were referred for endoscopic removal because this was often performed by other specialist endoscopists on dedicated programmes, possibly introducing selection bias (online supplementary table 4). Although optical diagnosis was recorded, participating endoscopists did not state if SMs or SMd was suspected. Besides, endoscopists were not formally trained in endoscopic recognition of the depth of invasion. On the other hand, endoscopists participating in this study were accredited for performing colonoscopies for the Dutch BCSP.^26^ We believe that the results of our study can therefore be extrapolated to other organised screening programmes in which general gastroenterologists perform colonoscopies.

10.1136/gutjnl-2018-316882.supp5 Supplementary data

In this prospective study, approximately two-thirds of the T1 CRCs were not recognised during consecutive FIT-positive colonoscopies for the Dutch BCSP, leading to suboptimal use of endoscopic and surgical treatment options. Training in structured lesion assessment is needed to improve endoscopic recognition of T1 CRCs and ensure optimal treatment strategies.

## References

[1] Vleugels JLA, Dijkgraaf MGW, Hazewinkel Y, et al Effects of training and feedback on accuracy of predicting rectosigmoid neoplastic lesions and selection of surveillance intervals by endoscopists performing optical diagnosis of diminutive polyps. Gastroenterology 2018;154:1682–93. 10.1053/j.gastro.2018.01.063 29425923

[2] Vleugels JLA Advanced endoscopic imaging for detection and differentiation of colorectal neoplasia (PhD-thesis. University of Amsterdam Digital Academic Repository (UvA-DARE), 2018.

[3] Hamilton SR, Aaltonen LA World Health organization classification of tumours. pathology and genetics of tumours of the digestive system. Lyon: IARC Press, 2010.

[4] Haggitt RC, Glotzbach RE, Soffer EE, et al Prognostic factors in colorectal carcinomas arising in adenomas: implications for lesions removed by endoscopic polypectomy. Gastroenterology 1985;89:328–36. 10.1016/0016-5085(85)90333-6 4007423

[5] Kikuchi R, Takano M, Takagi K, et al Management of early invasive colorectal cancer. risk of recurrence and clinical guidelines. Dis Colon Rectum 1995;38:1286–95. 10.1007/bf02049154 7497841

[6] Compton CC Colorectal carcinoma: diagnostic, prognostic, and molecular features. Mod Pathol 2003;16:376–88. 10.1097/01.MP.0000062859.46942.93 12692203

[7] Lugli A, Kirsch R, Ajioka Y, et al Recommendations for reporting tumor budding in colorectal cancer based on the International tumor budding consensus conference (ITBCC) 2016. Mod Pathol 2017;30:1299–311. 10.1038/modpathol.2017.46 28548122

[8] Toes-Zoutendijk E, Kooyker AI, Elferink MA, et al Stage distribution of screen-detected colorectal cancers in the Netherlands. Gut 2018;67:1745–6. 10.1136/gutjnl-2017-315111 29055907

[9] Belderbos TDG, van Erning FN, de Hingh IHJT, et al Long-Term recurrence-free survival after standard endoscopic resection versus surgical resection of submucosal invasive colorectal cancer: a population-based study. Clin Gastroenterol Hepatol 2017;15:403–11. 10.1016/j.cgh.2016.08.041 27609703

[10] Bosch SL, Teerenstra S, de Wilt JHW, et al Predicting lymph node metastasis in pT1 colorectal cancer: a systematic review of risk factors providing rationale for therapy decisions. Endoscopy 2013;45:827–41. 10.1055/s-0033-1344238 23884793

[11] Ricciardi R, Madoff RD, Rothenberger DA, et al Population-based analyses of lymph node metastases in colorectal cancer. Clin Gastroenterol Hepatol 2006;4:1522–7. 10.1016/j.cgh.2006.07.016 16979956

[12] Cooper GS, Xu F, Barnholtz Sloan JS, et al Management of malignant colonic polyps: a population-based analysis of colonoscopic polypectomy versus surgery. Cancer 2012;118:651–9. 10.1002/cncr.26340 21751204 PMC3193545

[13] Overwater A, Kessels K, Elias SG, et al Endoscopic resection of high-risk T1 colorectal carcinoma prior to surgical resection has no adverse effect on long-term outcomes. Gut 2018;67:284–90. 10.1136/gutjnl-2015-310961 27811313

[14] Borschitz T, Gockel I, Kiesslich R, et al Oncological outcome after local excision of rectal carcinomas. Ann Surg Oncol 2008;15:3101–8. 10.1245/s10434-008-0113-x 18719965

[15] Ikematsu H, Yoda Y, Matsuda T, et al Long-term outcomes after resection for submucosal invasive colorectal cancers. Gastroenterology 2013;144:551–9. quiz e14 10.1053/j.gastro.2012.12.003 23232297

[16] Ueno H, Mochizuki H, Hashiguchi Y, et al Risk factors for an adverse outcome in early invasive colorectal carcinoma. Gastroenterology 2004;127:385–94. 10.1053/j.gastro.2004.04.022 15300569

[17] Puig I, López-Cerón M, Arnau A, et al Accuracy of the narrow-band imaging international colorectal endoscopic classification system in identification of deep invasion in colorectal polyps. Gastroenterology 2019;156:75–87. 10.1053/j.gastro.2018.10.004 30296432

[18] Backes Y, Schwartz MP, Ter Borg F, et al Multicentre prospective evaluation of real-time optical diagnosis of T1 colorectal cancer in large non-pedunculated colorectal polyps using narrow band imaging (the optical study). Gut 2019;68:271–9. 10.1136/gutjnl-2017-314723 29298873

[19] Bronzwaer MES, Koens L, Bemelman WA, et al Volume of surgery for benign colorectal polyps in the last 11 years. Gastrointest Endosc 2018;87:552–61. 10.1016/j.gie.2017.10.032 29108978

[20] van Nimwegen LJ, Moons LMG, Geesing JMJ, et al Extent of unnecessary surgery for benign rectal polyps in the Netherlands. Gastrointest Endosc 2018;87:562–70. 10.1016/j.gie.2017.06.027 28713061

[21] Rönnow C-F, Elebro J, Toth E, et al Endoscopic submucosal dissection of malignant non-pedunculated colorectal lesions. Endosc Int Open 2018;6:E961–8. 10.1055/a-0602-4065 30083585 PMC6070376

[22] Kuellmer A, Mueller J, Caca K, et al Endoscopic full-thickness resection for early colorectal cancer. Gastrointest Endosc 2019;89:1180–9. 10.1016/j.gie.2018.12.025 30653939

[23] Burgess NG, Hourigan LF, Zanati SA, et al Risk Stratification for Covert Invasive Cancer Among Patients Referred for Colonic Endoscopic Mucosal Resection: A Large Multicenter Cohort. Gastroenterology 2017;153:732–42. 10.1053/j.gastro.2017.05.047 28583826

[24] Rutter MD, Chattree A, Barbour JA, et al British Society of Gastroenterology/Association of Coloproctologists of great britain and ireland guidelines for the management of large non-pedunculated colorectal polyps. Gut 2015;64:1847–73. 10.1136/gutjnl-2015-309576 26104751 PMC4680188

[25] Ferlitsch M, Moss A, Hassan C, et al Colorectal polypectomy and endoscopic mucosal resection (EMR): European Society of gastrointestinal endoscopy (ESGE) clinical guideline. Endoscopy 2017;49:270–97. 10.1055/s-0043-102569 28212588

[26] Bronzwaer MES, Depla ACTM, van Lelyveld N, et al Quality assurance of colonoscopy within the Dutch national colorectal cancer screening program. Gastrointest Endosc 2019;89:1–13. 10.1016/j.gie.2018.09.011 30240879

[27] Dekker E, Tanis PJ, Vleugels JLA, et al Colorectal cancer. Lancet 2019;394:1467–80. 10.1016/S0140-6736(19)32319-0 31631858

