# Supplementary material for: Suboptimal endoscopic cancer recognition in colorectal lesions in a national bowel screening programme
Source: Gut. 2019 Dec 10;69(6):977–80. doi: 10.1136/gutjnl-2018-316882 (PMC7282551; doi:10.1136/gutjnl-2018-316882)
Supplement: Supplementary data [file gutjnl-2018-316882supp001.pdf]

SUPPLEMENTAL MATERIAL 1

METHODS

Study design

This study was a post-hoc additional analysis of a prospective observational multicentre study in the Netherlands comparing optical diagnosis of diminutive (1-5mm) colorectal lesions using Narrow Band Imaging (NBI) with the outcomes of histopathology. The methods and results of the DISCOUNT 2 study have been reported in detail elsewhere. The current study is reported in line with the Standards for Reporting Diagnostic Accuracy (STARD) statement.

Study setting and training of participating endoscopists

The study was performed in patients undergoing colonoscopy after a positive FIT-result in the Dutch national BCSP with a cut off value of 47 µg/g feces (FOB gold, Sentinel, Milan, Italy). Individuals eligible for the national Dutch BCSP are aged between 55-75 years old, are biennially invited to participate in the screening program and all persons with a positive FIT result are invited for an precolonoscopy interview to discuss the test result and determine whether there are no contraindications for colonoscopy. Exclusion criteria for undergoing colonoscopy in the Dutch BCSP are a life-expectancy of less than five years, personal history of inflammatory bowel disease, previous proctocolectomy or current treatment for CRC.

The study was performed in 12 different regional hospitals and one academic hospital. All endoscopists were accredited for performing colonoscopies in FIT-positive patients for the Dutch BCSP. The accreditation-process included an e-learning on various aspects of colonoscopy. Furthermore, the knowledge and skills of the endoscopist in measuring current evidence-based quality indicators were tested, and practical skills during colonoscopy were evaluated. Procedures were performed with EVIS EXERA II or III video processors (Olympus, Tokyo, Japan) and "180"- or "190"-colonoscopes (Olympus, Tokyo, Japan) including NBI.

Endoscopists attended one of the three training meetings organised in January 2015 and received our previously validated training in the Workgroup serrAted polypS and Polyposis (WASP) classification.[27] The WASP classification combines NBI International Colorectal Endoscopic classification (NICE) and specific criteria for sessile serrated lesions (SSLs) to differentiate between adenomas, hyperplastic polyps (HPs) and SSLs. Although this training primarily aimed to educate participants in differentiating diminutive lesions, it also included five examples of the Kudo classification type V and type 3. After this training, endoscopists were required to perform a test based on still white-light and corresponding NBI images of 1-9mm adenomas, HPs and SSLs. Participating endoscopists completed the test if they achieved at least 90% accuracy in differentiating between neoplastic (adenomas and SSLs) and non-neoplastic polyps (HPs). Once passed, a real-time test-phase was conducted in consecutive colonoscopies for the Dutch BCSP. For at least 20 diminutive or small polyps, optical diagnosis should result in achieving ≥90% accuracy in differentiating between neoplastic and non-neoplastic polyps. In total, 27 endoscopists achieved both tests and recorded optical diagnosis of all detected lesions for 1 year during consecutive FIT-positive colonoscopies. All FIT-positive colonoscopies where optical diagnoses were recorded were included in this additional analysis.

Data-collection

In each participating centre, data were anonymously collected from electronic medical records, endoscopy and pathology reports. Patient characteristics included age in years, gender and American Society of Anesthesiologists (ASA) classification. For patients with suspected or established CRC, additional information on follow-up colonoscopies, outpatient clinic visits, adjuvant local or surgical therapy and histopathological outcomes of adjuvant therapy were collected. Endoscopic treatment and TEM were defined as local treatment.

During the study, endoscopists used a standard colonoscopy-reporting system for the Dutch BCSP with an obligatory choice of fixed text-blocks. This facilitated high-quality data-collection and ensured collecting exact data on all aspects of each detected lesion as location, size, Paris morphology, optical diagnosis (CRC, adenoma, HP, SSL or other), endoscopic treatment method, completeness of resection and whether a tattoo was placed. For the purpose of this study, endoscopists recorded an optical diagnosis including a confidence level for all detected lesions irrespective of its size. Endoscopists were asked to predict lesion histology using both white-light imaging and NBI. Treatment characteristics included method of resection (biopsied, cold or hot snare polypectomy, endoscopic mucosal resection, biopsy for diagnosis or no treatment) and whether the resection was performed en bloc or in piecemeal.

For the Dutch screening program, pathologists reported all histological parameters in a standardised and structured fashion, facilitating complete data collection. Histological parameters included the size determined by the pathologist, histological assessment including grade of dysplasia and assessment of resection margins. In case of cancer, histological differentiation grade of the tumor, depth of invasion, presence or absence of lymphovascular invasion and assessment of resection margin was reported.

All data were entered in an online database (CastorEDC) by the study coordinator. CastorEDC (http://www.castoredc.com) is a good clinical practice approved secure, web-based and user-friendly application for data collection and storage for research studies.

Histopathology and histopathology revision

All resected lesions were collected in separate containers and were assessed by a pathologist with expertise in gastrointestinal pathology in the local hospital. Prior to the start of the Dutch BCSP, these pathologists underwent an accreditation process in order to standardize quality of histopathological assessment of colonic lesions. During this accreditation process, pathologists had to complete an obligatory online e-learning module (http://bevolkingsonderzoek.bash01.nl/) that consisted of several modules, including a separate module regarding histological classification of T1 CRCs. After completing this training module, pathologists were required to perform an examination with 50 diagnostic slides. To become accredited, a score of at least 90% was required. Histologic assessment was performed according to the 2010 WHO classification.

From all patients initially diagnosed with T1 CRCs, the original hematoxylin-eosin staining slides were collected from local hospitals. A single specialist gastrointestinal pathologist (LK) performed a blinded pathology review on the original hematoxylin-eosin staining slides to provide complete histological information on all T1 CRCs. In case of disagreement between the initial diagnosis and expert review, another specialist gastrointestinal pathologist was consulted. Final outcome was based on agreement between the two specialist pathologists. During pathology revision, the grade of tumor differentiation was assessed according to the 2010 WHO classification. Depth of invasion was assessed according the Haggitt classification for pedunculated lesions and Kikuchi classification for non-pedunculated lesions. For non-pedunculated lesions, invasion depth ≥1,000 µm (Kikuchi level sm2⁄3) was defined as deep submucosal invasion. For lymphovascular invasion, the pathologist evaluated

whether cancer cells were present within endothelial-lined channels. Finally, the grade of tumor budding was defined according to the most recently published consensus report.

Study outcomes

The primary outcome of this study was to 1) determine the rate of correct optical diagnoses of T1 CRCs and 2) to compare the treatment approaches for T1 CRCs with a correct optical diagnosis of cancer versus those that were not correctly diagnosed. Secondary study outcomes included the diagnostic test accuracy (i.e. sensitivity, specificity and predictive values) for the endoscopic diagnosis of cancer. Furthermore, false positive and false negative optical diagnosis, including treatment and histopathology outcomes, were described.

Statistical analysis

Categorical variables were expressed as counts and corresponding percentages, continuous normally distributed data as means including their standard deviation (SD) or medians with their interquartile ranges (IQRs) in case of skewed data. Proportions were compared with the $\chi 2$ test or Fisher exact test. For comparisons of normally distributed data, the t-test was used, while for non-parametric data, the Mann-Whitney U test was used.

The following analyses were performed on a per-lesion basis. Lesions were excluded from the analysis if no endoscopic histology prediction (index test) or histopathology result (reference test) was available. Lesions that were referred for treatment evaluation during a follow-up colonoscopy were also excluded from the analysis as these exams were performed by specialist endoscopists on dedicated programs. To determine the number of true positives, false positives, true negatives and false negatives for the optical diagnosis of CRC, two by two contingency tables were created. These two by two contingency tables were also used to calculate sensitivity, specificity and predictive values and their 95% confidence intervals (CIs). These estimates were calculated by comparing the optical diagnosis by the endoscopist with the histopathology findings, which was used as the reference standard. For both the endoscopic diagnosis as histology outcome, lesions were categorised as CRC or non-CRC (adenoma, SSL and HP or other not including CRC).

SPSS version 24.0 (IBM SPSS Statistics for Windows, Armonk, NY, USA) and R version 3.4.3 (The R Foundation for Statistical Computing, Vienna, Austria) was used for analysis. Diagnostic test accuracies were calculated using the epiR package. A two-sided p value <0.05 was considered statistically significant.

Ethical approval and role of the funding source

The Institutional Review Board of the Academic Medical Centre decided that formal revision was not required according to the Medical Research Involving Human Subjects Act (WMO) because patient data were retrieved during standard care without any interventions (W14_099#14.17.0127). The study protocol was approved by the Institutional Review Board of each participating centre. Participating endoscopists provided written informed consent as they were regarded the study subjects. All patient data was coded and anonymity of patients was guaranteed. All authors had access to the study data and all authors reviewed and approved the final manuscript. The DISCOUNT 2 study was preregistered (*NCT02407925, NTR4635*) and funded by the Dutch Digestive Disease Foundation (*FP13-10*).
