# Supplementary material for: Suboptimal endoscopic cancer recognition in colorectal lesions in a national bowel screening programme
Source: Gut. 2019 Dec 10;69(6):977–80. doi: 10.1136/gutjnl-2018-316882 (PMC7282551; doi:10.1136/gutjnl-2018-316882)
Supplement: Supplementary data [file gutjnl-2018-316882supp002.pdf]

SUPPLEMENTAL TABLE 1.

Characteristics of FIT-positive patients diagnosed with cancer.

		**Patients (N = 274)**
Female gender, n (%)		126 (46%)
Age, median (IQR)		67 (63-69)
ASA classification, n (%)		
	1	69 (25%)
	2	186 (68%)
	3	11 (4%)
	Missing	8 (3%)
Anticoagulant medication, n (%)		71 (26%)
T stage cancer, n (%)		
	T1	90* (40%)
	T2	59 (26%)
	T3	69 (30%)
	T4	9 (4%)
N stage cancer, n (%)		
	Nx or N0	163 (50%)
	N1	44 (15%)
	N2	20 (7%)
M stage cancer, n (%)		
	Mx or M0	217 (96%)
	M1	10 (4%)
TNM classification missing, n (%)		
	Neo-adjuvant treatment	23 (8%)
	Referred other center	19 (7%)
	Died before staging	3 (1%)
	Diagnosed with lymphoma	2 (1%)

*Two patients were diagnosed with 2 T1 CRCs. N=number, IQR=interquartile range, ASA=American Society of Anaesthesiologists, TNM=TNM classification of malignant tumours
