# Supplementary material for: Suboptimal endoscopic cancer recognition in colorectal lesions in a national bowel screening programme
Source: Gut. 2019 Dec 10;69(6):977–80. doi: 10.1136/gutjnl-2018-316882 (PMC7282551; doi:10.1136/gutjnl-2018-316882)
Supplement: Supplementary data [file gutjnl-2018-316882supp003.pdf]

SUPPLEMENTAL TABLE 2.

Outcomes of low and high confidence optical diagnosis.

**High confidence (N=7,837)**		
**Diagnostic test accuracy**	**% (95% CI)**	**True proportion**
Sensitivity	71.3% (63.8-78.0)	119 / 167
Specificity	99.9% (99.7-99.9)	7,661 / 7,672
Positive predictive value	91.5% (85.6-95.2)	119 / 130
Negative predictive value	99.4% (99.2-99.5)	7,661 / 7,709
**Low confidence (N=1,753)**		
**Diagnostic test accuracy**	**% (95% CI)**	**True proportion**
Sensitivity	54.2% (32.8-74.5)	13 / 24
Specificity	98.8% (98.2-99.3)	1,708 / 1,729
Positive predictive value	38.2% (26.1-52.1)	13 / 34
Negative predictive value	99.4% (99.0-99.6)	1,708 / 1,719

*Low or high confidence optical diagnosis was not recorded for 411 optical diagnoses
