# Supplementary material for: Suboptimal endoscopic cancer recognition in colorectal lesions in a national bowel screening programme
Source: Gut. 2019 Dec 10;69(6):977–80. doi: 10.1136/gutjnl-2018-316882 (PMC7282551; doi:10.1136/gutjnl-2018-316882)
Supplement: Supplementary data [file gutjnl-2018-316882supp004.pdf]

SUPPLEMENTAL TABLE 3.

Results of endoscopic and surgical management of T1 CRCs stratified for pedunculated versus non-pedunculated morphology.

	**Non-pedunculated T1 CRCs**			**Pedunculated T1 CRCs**		
	**Optical diagnosis CRC (N=26)**	**Optical diagnosis benign (N=33)**		**Optical diagnosis CRC (N=10)**	**Optical diagnosis benign (N=23)**	
**Primary local* treatment, (N=72)**						
	8 (31%)	31 (94%)		10 (100%)	23 (100%)	
Local treatment method, n (%)						
En bloc endoscopic	5 (63%)	21 (68%)		10 (100%)	22 (96%)	
Piecemeal endoscopic	-	10 (32%)		-	1 (4%)	
TEM	3 (37%)	-		-	-	
Microscopic resection margin free of CRC (≥1mm) after local treatment, n (%)						
Yes	7 (80%)	14 (45%)		10 (100%)	20 (87%)	
No / unable to determine	1 (20%)	17 (55%)		-	3 (13%)	
Adjuvant TEM/surgical treatment after local treatment, n (%)						
TEM	1 (13%)	4 (12%)		-	-	
Surgical treatment	1 (12%)	14 (42%)		1 (10%)	8 (35%)	
Reason adjuvant TEM/surgical treatment after local treatment, n (%)						
Positive resection margin	1 (50%)	7 (35%)		-	2 (25%)	
Lymphovascular invasion	1 (50%)	1 (6%)		1 (100%)	4 (50%)	
Piecemeal removal	-	10 (59%)		-	1 (12.5%)	
Serrated polyposis syndrome	-	-		-	1 (12.5%)	
Adjuvant sigmoidoscopy/colonoscopy for tattoo placement, n (%)						
	-	21 (64%)		1 (11%)	16 (70%)	
**Primary surgical treatment, (N = 20)**						
	18 (69%)	2 (6%)		-	-	

*local treatment was defined as endoscopic removal and transanal endoscopic microsurgery. TEM= transanal endoscopic microsurgery
