# Supplementary material for: Suboptimal endoscopic cancer recognition in colorectal lesions in a national bowel screening programme
Source: Gut. 2019 Dec 10;69(6):977–80. doi: 10.1136/gutjnl-2018-316882 (PMC7282551; doi:10.1136/gutjnl-2018-316882)
Supplement: Supplementary data [file gutjnl-2018-316882supp005.pdf]

SUPPLEMENTAL TABLE 4.

Outcomes of referral colonoscopy of 191 patients with 208 lesions.

**Intervention**	**Number of patients**	**Reason referral**	**Histology outcome most advanced lesion**	**TNM stage**
Referred for removal of multiple adenomas	N=41	n.a.	HGD=7<br>LGD=34	
Referred for EMR	N=46	n.a.	CRC=9<br>HGD=11<br>LGD=25<br>Non-neoplastic=1	T1NxM0=7<br>T1N0M0=1<br>T1N1M0=1
Referred for pEMR	N=73	n.a.	CRC=3<br>HGD=7<br>LGD=59<br>SSL=4	T1N0M0=3
Referred for TEM	N=8	Size=8	HGD=4<br>LGD=4	
Referred ESD	N=1	Rectal location=1	LGD=1	
Referred eFTR	N=1	Involvement appendix=1	HGD=1	
Referred for surgical treatment	N=14	Unremovable=6<br>Suspicion CRC=3<br>Non-lifting sign=5	CRC=6<br>HGD=3<br>LGD=4<br>Fibroma=1	T1N0M0=5<br>T3N0M0=1
Missing	N=25			

LGD=low-grade dysplasia, HGD=high-grade dysplasia, EMR=endoscopic mucosal resection, T1 CRC=T1 colorectal cancer, pEMR=piecemeal endoscopic mucosal resection, SSL=sessile serrated lesion, TEM= transanal endoscopic microsurgery, ESD=endoscopic submucosal dissection, eFTR=endoscopic full thickness resection.
